# Study on the correlation between Left Ventricular Hypertrophy and Coronary Artery disease in the very elderly patients with hypertension

**DOI:** 10.12669/pjms.37.5.4135

**Published:** 2021

**Authors:** Jian-hua Sun, Xiao-kun Liu, Qi Zhang, Qing-hua Zhang

**Affiliations:** 1 Jian-hua Sun, Department of Cardiology, Tangshan Workers’ Hospital, Tangshan, Hebei, P.R. China; 2 Xiao-kun Liu, Department of Cardiology, Tangshan Workers’ Hospital, Tangshan, Hebei, P.R. China; 3 Qi Zhang, Department of Cardiology, Tangshan Workers’ Hospital, Tangshan, Hebei, P.R. China; 4 Qing-hua Zhang Department of Cardiology, Tangshan Workers’ Hospital, Tangshan, Hebei, P.R. China

**Keywords:** Coronary heart disease, Left ventricular hypertrophy, Very elderly

## Abstract

**Objective:**

To investigate the relationship between left ventricular hypertrophy (LVH) and coronary artery disease in the very elderly (over 80 years old) patients with hypertension.

**Methods:**

One hundred twenty cases of very elderly patients with hypertension admitted to our hospital from March 2018 to December 2020 were selected and divided into two groups: the LVH group and the non-LVH group, all of whom were older aged over 80 years, including 62 patients in the LVH group and 58 patients in the non-LVH group. All patients underwent cardiac color Doppler ultrasound examination, 24-hour dynamic ECG examination, and coronary angiography or coronary CTA examination. The clinical data of the two groups were analyzed statistically.

**Results:**

There were significant differences in the number of diseased vessels, degree of coronary stenosis and vascular calcification between the two groups (P<0.05). Moreover, the results of risk factors for the degree of coronary artery disease in the two groups showed that the history of diabetes, 2hPG and LVH were independent risk factors for the three-vessel disease, while the history of LVH, FPG and alcohol intake were independent risk factors for diffuse lesions, but there was no statistical difference in the correlation between them and the degree of coronary stenosis.

**Conclusion:**

LVH is an independent risk factor for coronary artery stenosis and calcification in the very elderly patients with hypertension, but there is no statistical difference in the correlation between LVH and the degree of coronary stenosis.

## INTRODUCTION

Hypertension has been proved by various studies to be an important cause of coronary heart disease. Elderly patients with hypertension are accompanied by LVH, increased myocardial weight, and myocardial remodeling, which may easily lead to congestive heart failure and other complications.^1^ LVH and diastolic dysfunction are important links in the pathogenesis and one of the independent risk factors for the onset of hypertension. It has been reported that the reduction in ejection fraction associated with hypertension has a close bearing on the total burden of myocardial fibrosis. In the event of excessive pressure overload (hypertension) duration, irreversible fibrosis of the left ventricle will occur, resulting in permanent systolic function impairment.^2^ Coronary heart disease has a high incidence in the elderly^3^, and it is clinically manifested as myocardial hypoxia, myocardial ischemia, infarction and other symptoms. It has been shown in most studies that hypertensive patients with coronary artery disease have a significantly higher incidence of increased left ventricular volume compared with patients with simple hypertension. In patients with complete occlusion of coronary artery, the degree of myocardial hypertrophy was significantly increased, indicating a certain relationship between myocardial remodeling and the degree of coronary artery lesions.^4^

China has gradually stepped into an aging society, and the number of the very elderly (currently over 80 years old according to international regulations) has increased significantly. In view of this, myocardial hypertrophy and coronary artery disease in the very elderly population were studied by our hospital to try to explore the correlation between the two diseases.

## METHODS

One hundred and twenty cases of the very elderly patients with hypertension admitted to Tangshan Workers’ Hospital from March 2018 to December 2020 were included and divided into two groups after echocardiography by an ultrasonic diagnostic equipment^5^: the LVH group and the non-LVH group, with an age of 85 ± 5 years, of which 62 patients were in the LVH group and 58 patients were in the non-LVH group. All patients underwent coronary angiography and were diagnosed with coronary heart disease or coronary artery disease.

### Ethical approval

The study was approved by the Institutional Ethics Committee of Tangshan Workers’ Hospital at December 8, 2020, and written informed consent was obtained from all participants.

### Exclusion criteria:

1. Patients with cardiomyopathy, severe arrhythmia, myocardial infarction, or heart failure.
2. Patients with liver, kidney and mental diseases.
3. Patients with tumors and digestive tract diseases.

No significant difference can be found between the two groups in terms of age, sex, length of hospital stays and other general information (P<0.05).

Both groups of patients underwent cardiac color Doppler ultrasound examination.^6^ The patient under examination was in the left lateral decubitus position, and the GE-E90 ultrasound instrument, with its probe frequency set to 2.5 - 4.0 MHz, was placed at the apex to detect the left atrial diameter, myocardial mass, end-diastolic volume, end-systolic volume, left ventricular ejection fraction, ratio of peak E to peak A of the end-diastolic blood flow velocity of the mitral valve orifice and other cardiac function indicators.

When performing coronary angiography, the radial artery was selected as the approach by the Seldinger method, and left and right coronary angiography was performed by the Judkins method in multiple angles and positions. Selective coronary angiography with one or more coronary artery diameter stenosis I > 50% was used as the diagnostic criteria for coronary heart disease. Coronary CTA was examined by Siemens’ new dual-source CT. The degree of coronary artery disease is manifested in the following aspects^7^,^8^:

1) Patients with coronary heart disease were classified into single-vessel, double-vessel, and multiple-vessel lesions according to the number of lesions involved in the left anterior descending artery, left circumflex artery and right coronary artery.Diffuse lesions of coronary arteries, including: length of lesions greater than 20 mm; all or most of the tortuous, loosely spring-like blood vessels; multiple lesions in one vessel. The degree of coronary artery stenosis was divided into: moderate stenosis, 50%-74% of diseased vascular stenosis; severe stenosis, 75%-99% of diseased vascular stenosis; complete occlusion, 100% of diseased vascular stenosis.

### Statistical Analysis

All the data were statistically analyzed by SPS22.0 software, and the measurement data were expressed as (x^2^±s). Two independent sample t-test and standard analysis of variance were used for inter-group data analysis, and the univariate analysis was used for comparison of mean values between the two groups. P<0.05 indicates a statistically significant difference.

## RESULTS

The comparison of the number of diseased vessels between the two groups and the comparison of the formation of single-vessel, two-vessel, and three-vessel disease between the two groups are shown in Table-I, with significant differences (P<0.05).The comparison of vascular distribution between the two groups, and the comparison of left anterior descending (LAD), left circumflex artery (LCX), right coronary artery (RCA) and left main coronary artery (LM) are also shown in Table-I.

**Table-I:** Comparison of the number of diseased vessels, degree of stenosis, and diseased vessels between the two groups [n (%)].

Group	Number of cases	Number of diseased vessels			Degree of stenosis			Diffuse disease	Diseased vessel			

		Single-vessel	Two-vessel	Three-vessel	Moderate	Severe	Complete occlusion		LAD	LCX	RCA	LM
LVH group	62	18 (29%)	29 (46.7%)	15 (24.2%)	22 (35.5%)	34 (548%)	6 (9.7%)	25 (40.3%)	34 (54.8%)	25 (40.3%)	31 (50%)	10 (16.1%)
Non-LVH group	58	32 (55.2%)	22 (37.9%)	4 (6.9%)	33 (56.9%)	24 (39.7%)	1 (3.4%)	12 (20.7%)	17 (29.3%)	14 (24.1%)	21 (36.2%)	4 (6.9%)
X^2^			23.31			25.54		15.23	16.32	13.65	12.54	21.21
*P*			<0.02			<0.01		0.03	0.04	0.03	0.03	<0.01

There were significant differences in the degree of vascular lesions and the proportion of moderate stenosis, severe stenosis and complete occlusion between the two groups (P<0.05). In addition, there was a significant difference in the diffuse incidence rate between the two groups (P<0.05) Table-I.

*In terms of comparative analysis of the risk factors of coronary artery disease in the two groups*, age, time of onset, body mass index, history of alcohol intake, history of diabetes, fasting blood glucose (FPG), 2-hour postprandial blood glucose (2hPG), glycosylated hemoglobin (HbA1c), serum total cholesterol (TC), and low-density lipoprotein (LDL-C) were selected as independent variables, and three-vessel disease, diffuse disease, and severe stenosis were used as dependent variables for logistic regression analysis. The results show that history of diabetes and 2hPG were independent risk factors for three-vessel disease, FPG and history of alcohol intake were independent risk factors for diffuse disease, and severe stenosis was an independent risk factor for serum total cholesterol (TC) Table-II.

**Table-II:** Risk factors for the degree of coronary artery disease.

Type of disease	Independent variable	b	OR value	95% CI		*P*-value
Three-vessel disease	History of diabetes	0.915	2.134	1.103	5.478	0.032
	2hPG	1.435	3.457	1.564	8.375	0.009
Diffuse disease	HbA_1c_	1.367	3.238	1.456	7.998	0.012
	History of alcohol intake	1.769	4.121	1.982	13.231	0.003
Severe stenosis	TC	0.712	2.013	1.015	7.235	0.041

## DISCUSSION

Hypertension is an important indicator of coronary heart disease, and ventricular hypertrophy has been proved by research data to have a certain role in promoting the development of coronary heart disease.^9^ In patients with hypertension, the period of high pressure reaches 160-190mmhg and low pressure reaches 100-109 is clinically characterized by LVH and organic diseases in the heart, brain and kidney system.^10^,^11^ The heart and blood vessels are the main target organs for the pathophysiological effects of hypertension. Hypertensive heart disease, characterized by symmetrical hypertrophy, asymmetric hypertrophy and extendible hypertrophy, is known as hypertensive heart disease due to cardiac hypertrophy and expansion caused by long-term high pressure load, cardiomyocyte hypertrophy and interstitial fibrosis changes.^12^-^14^ Such a hypertensive heart disease is often accompanied by coronary heart disease and microvascular disease, which may eventually lead to heart failure, arrhythmia, and even sudden death.^15^ Under normal circumstances, patients with coronary heart disease will have one or more coronary arteries with a certain degree of stenosis because patients with long-term hypertension will have changes in the wall of blood vessels and prone to arteriosclerosis, leading to coronary heart disease. In this study, the subjects are divided into two groups: the LVH group and the non-LVH group. In terms of the number of diseased arteries, the number of single-vessel disease in the non-LVH group is greater than that in the LVH group, and the number of multi-vessel disease in the non-LVH group is less than that in the LVH group, with significant differences. Patients in the LVH group are basically able to reach the Phase II of hypertension, and the organic diseases of the organs in the physiological tissue lesions have caused functional damage to the local blood vessels. Most of the patients in the non-LVH group are in Phase I of hypertension and have no obvious pathological features. It can be found from the degree of vascular stenosis that the proportion of complete vessel occlusion in the LVH group is 9.7%, while that in the non-LVH group is 3.4%. There is a significant difference between the two groups. In the proportion of patients with severe vascular occlusion, the proportion of patients in the LVH group (56.9%) is greater than that in the non-LVH group (35.5%), further indicating the severity of the disease in the LVH group. In terms of the proportion of diseased vessels, LM is the most important vessel in the heart. The incidence of LM in patients with LVH (16.1%) is significantly higher than that in the non-LVH group (6.9%), and the incidence of other three vessels in the LVH group is also higher than that in the non-LVH group, with significant differences (P<0.05). Arteriosclerosis is mainly manifested as hardening of arterial vessel walls and loss of elasticity, narrowing of lumen, resulting in less compliance, weakening of arterial elasticity, higher systolic blood pressure and lower diastolic blood pressure.^16^ Coupled with the increased mechanical scouring effect of blood flow on the vessel wall under hypertension, the blood pressure of patients with stage II hypertension (mostly patients in the LVH group) is significantly higher than that of patients with stage I hypertension (mostly patients in the non-LVH group). Such a scouring force increases the permeability of the intima to lipids so that lipid proteins can penetrate the intima, monocytes migrate to the intima via adhesion, or platelets migrate to the intima, all of which can cause atherosclerosis of arteries.

The degree of coronary artery disease also showed a yearly increase with age, and its incidence is also associated with certain independent risk factors. According to our study, age, time of onset, body mass index, history of alcohol intake, history of diabetes, fasting blood glucose (FPG), 2-hour postprandial blood glucose (2hPG), glycosylated hemoglobin (HbA1c), serum total cholesterol (TC), and low-density lipoprotein (LDL-C) were first established as independent variables, and three-vessel disease, diffuse disease, and severe stenosis were selected as dependent variables, and then independent risk factor analysis of coronary arteries was performed. The results show that history of diabetes and 2hPG are independent risk factors for three-vessel disease, indicating that blood glucose affects the blood viscosity to a certain extent, and embolization is more likely to be formed for the damaged vessels, thus inducing the occurrence and development of three-vessel disease. With the increase of age, especially for the very elderly patients, the increase of age leads to the deterioration of physical skills, the increase of the probability of coronary artery branch disease, and the corresponding increase in the complexity of the disease.^17^ It is shown in our study that history of alcohol intake and HbA1c are independent risk factors for diffuse disease. Most patients who drink alcohol will have blood circulatory disorders due to the long-term effects, and the accumulation of platelets will lead to the formation of thrombus.^18^ HbA1c > 9% indicates that patients with persistent hyperglycemia, which is easy to develop into diseases such as arteriosclerosis. It has been shown in a large number of studies that cardiovascular diseases of different degrees will occur if the proportion of HbA1c is greater than 7.5% in patients with LVH. The content of total serum cholesterol (TC) is an independent risk factor for severe stenosis, which is consistent with previous literature reports.^19^,^20^

## CONCLUSION

The incidence of coronary heart disease in the very elderly hypertensive patients with LVH is increased. It is found that the history of diabetes, 2hPG, and LVH are independent risk factors for three-vessel disease, LVH, FPG, and history of alcohol intake are independent risk factors for diffuse coronary disease according to the comparative study of independent correlations between the onset of ventricular hypertrophy and non-ventricular hypertrophy in the very elderly patients, but there was no statistical difference in the correlation between them and the degree of coronary stenosis. Therefore, early control of blood pressure and long-term control of blood pressure to reach the standard may reduce the incidence and severity of left ventricular hypertrophy and coronary heart disease.

### Authors’ Contributions:

**JHS and XKL** designed this study and prepared this manuscript, and are responsible and accountable for the accuracy or integrity of the work.

**QZ** collected and analyzed clinical data.

**QHZ** significantly revised this manuscript.
